# GSA CONGRESSIONAL UPDATE

**DOI:** 10.1093/geroni/igae098.1517

**Published:** 2024-12-31

**Authors:** Patricia D’Antonio

**Affiliations:** Gerontological Society of America, Washington, District of Columbia, United States

## Abstract

This popular annual session will provide cutting-edge information on what the 118th Congress has and has not accomplished to date related to aging policy. Of particular importance will be the Older Americans Act Reauthorization, including funding for the Center for Research and Demonstration in the Aging Network, as well as an update on Medicare Advantage, nursing home regulations, and hospice and palliative care legislation.

